# Comparable Ages for the Independent Origins of Electrogenesis in African and South American Weakly Electric Fishes

**DOI:** 10.1371/journal.pone.0036287

**Published:** 2012-05-14

**Authors:** Sébastien Lavoué, Masaki Miya, Matthew E. Arnegard, John P. Sullivan, Carl D. Hopkins, Mutsumi Nishida

**Affiliations:** 1 Institute of Oceanography, National Taiwan University, Taipei, Taiwan; 2 Atmosphere and Ocean Research Institute, The University of Tokyo, Chiba, Japan; 3 Department of Zoology, Natural History Museum & Institute, Chiba, Japan; 4 Human Biology Division, Fred Hutchinson Cancer Research Center, Seattle, Washington, United States of America; 5 Cornell University Museum of Vertebrates, Ithaca, New York, United States of America; 6 Department of Neurobiology and Behavior, Cornell University, Ithaca, New York, United States of America; Texas A&M University, United States of America

## Abstract

One of the most remarkable examples of convergent evolution among vertebrates is illustrated by the independent origins of an active electric sense in South American and African weakly electric fishes, the Gymnotiformes and Mormyroidea, respectively. These groups independently evolved similar complex systems for object localization and communication via the generation and reception of weak electric fields. While good estimates of divergence times are critical to understanding the temporal context for the evolution and diversification of these two groups, their respective ages have been difficult to estimate due to the absence of an informative fossil record, use of strict molecular clock models in previous studies, and/or incomplete taxonomic sampling. Here, we examine the timing of the origins of the Gymnotiformes and the Mormyroidea using complete mitogenome sequences and a parametric Bayesian method for divergence time reconstruction. Under two different fossil-based calibration methods, we estimated similar ages for the independent origins of the Mormyroidea and Gymnotiformes. Our absolute estimates for the origins of these groups either slightly postdate, or just predate, the final separation of Africa and South America by continental drift. The most recent common ancestor of the Mormyroidea and Gymnotiformes was found to be a non-electrogenic basal teleost living more than 85 millions years earlier. For both electric fish lineages, we also estimated similar intervals (16–19 or 22–26 million years, depending on calibration method) between the appearance of electroreception and the origin of myogenic electric organs, providing rough upper estimates for the time periods during which these complex electric organs evolved *de novo* from skeletal muscle precursors. The fact that the Gymnotiformes and Mormyroidea are of similar age enhances the comparative value of the weakly electric fish system for investigating pathways to evolutionary novelty, as well as the influences of key innovations in communication on the process of species radiation.

## Introduction

The Mormyroidea and Gymnotiformes, the African and South American weakly electric fishes respectively, have been the object of neuroethological research for decades (e.g. [1]–[10]), and are emerging as excellent comparative vertebrate systems for evolutionary neurobiology. Recent work on these two groups has investigated mechanisms by which animal communication and associated nervous system functions evolve and have feedback effects on evolutionary processes. Examples include studies of reproductive character displacement [11], the role of communication in speciation [12]–[15], effects of the evolution of neural structures on the process of species radiation [16]–[18], and genetic mechanisms underlying the origins of evolutionary novelty [19], [20]. Although these areas of investigation are informed by recent phylogenetic advances [19], [21]–[24], inferences about the temporal context of these processes are limited by the lack of an hypothesis for the timing of the origin and early diversification of these two groups of electrogenic teleosts. The purpose of this paper is to hypothesize just such a phylogenetic timeframe for the African and South American weakly electric fishes and to discuss its implications for understanding their evolution.

### Extraordinary Convergences Around a Novel Sensory and Communication System

**Figure 1:**
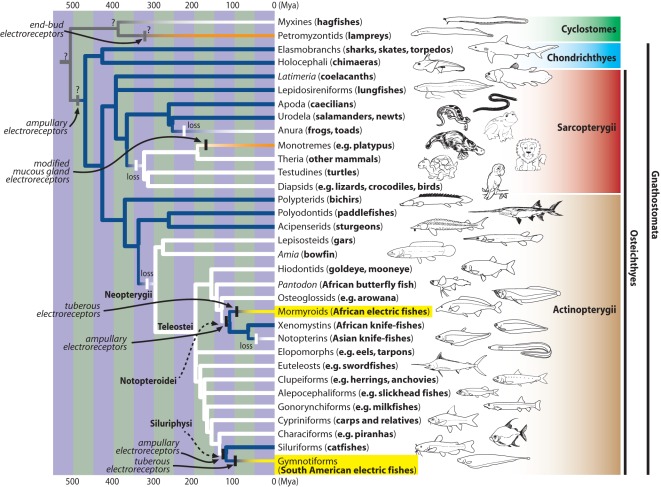
Phylogenetic distribution of electroreception within the Craniata and its evolution according to the criterion of parsimony. The phylogenetic backbone shown here follows Nelson [140], with modifications according to Gardiner et al. [141], Lavoué et al. [119], [142], Heimberg et al. [143], Kikugawa et al. [144], Li et al. [120], and Takezaki et al. [145]. Approximate timeline adapted from the fossil record; data on electroreception and electroreceptors taken from Bullock et al. [1], [26] and Albert and Crampton [25]. Colored branches indicate electroreceptive lineages possessing electroreceptors: as modified mucous glands (orange); of the ampullary sense organ type (deep blue); of both the tuberous sense organ type and the ampullary sense organ type found in teleosts (yellow). White branches signify non-electroreceptive lineages following secondary loss of electroreceptive capability; four (possibly five) such losses are indicated by white hash marks. The origins of different forms of electroreception are indicated by black hash marks. The electroreceptive conditions of the ancestors of the Craniata and of the clade (hagfishes, lampreys) are unresolved (indicated with grey and question marks) because there are several equi-parsimonious hypotheses concerning them. The end bud electroreceptor of the lampreys and the ampullary electroreceptor of the basal gnathostomes are anatomically very different, suggesting independent origins. The tree does not map atypical reports of electroreceptive gains in single species, which are in need of further study, such as tuberous electroreceptors in a blind catfish [146]. Recently, Czech-Damal et al. [147] discovered a novel sensory organ and possible electroreceptors associated with the hairless vibrissal crypts on the snout of the Guiana Dolphin (*Sotalia guianensis*), which appear to be sensitive to weak D.C. electric fields on the order of 4.6 microvolts per cm. Although their studies so far involve only one captive specimen trained to respond to the presence or absence of weak electric fields, it indeed suggests that additional research is needed on the sensory capabilities of aquatic mammals that might have independently evolved electroreception. Piranha (*Catoprion mento*) and platypus illustrations modified from images downloaded from Wikimedia Commons; paddlefish (*Polyodon spathula*) illustration modified from NOAA’s Historic Fisheries Collection Catalog of Images; other fish illustrations modified from Nelson [140]; other tetrapod illustrations taken from Léo Lavoué’s coloring book.

Electroreception, the ability to sense weak electric fields, is widely distributed in non-teleost aquatic craniates (Fig. 1). Ampullary electroreceptors, which are tuned to passively produced, low frequency electric fields, are found in lineages ranging from jawless craniates (lampreys) to several groups of “ancient fishes” such as chondrichthyans, coelacanths, and sturgeons [1], [25], [26]. This pattern suggests that electroreception is an ancient sense within the Craniata. However, because several hypotheses concerning reconstruction of the evolution of electroreception are equally parsimonious, it cannot be determined whether the most recent common ancestor of all craniates was electroreceptive (Fig. 1). Within teleost fishes–by far the largest group of vertebrates with more than 31,000 species [27]–electroreception is restricted to only two distantly related groups: the Siluriphysi *sensu* Fink and Fink [28] (i.e., the Gymnotiformes plus Siluriformes) and the Notopteroidei (i.e., the Mormyroidea plus Notopteridae). The most parsimonious hypothesis for this peculiar pattern is that low frequency electroreception was lost in the most recent common ancestor of the Neopterygii, only to be independently re-acquired in the Siluriphysi and the Notopteroidei (Fig. 1). Whereas the African Notopteridae and Siluriformes are only passively electroreceptive, the Gymnotiformes and the Mormyroidea secondarily and independently evolved specialized electric organs dedicated to the production of weak electric discharges, in addition to high-frequency (tuberous) electroreceptors that are tuned to these signals. Together, their electric organs and tuberous electroreceptors mediate both electrical communication and “active electrolocation” (e.g. [29]), in which objects are located in space and their electrical properties sensed via distortions in the self-generated electric field [30]. Thus, the ability to first sense low frequency, passive electric fields appears to have preceded the evolution of electrogenesis in the Gymnotiformes and Mormyroidea [31], [32].

All mormyroids and gymnotiforms except *Electrophorus electricus* are referred to as “weakly” electric fishes, because the external potentials they produce are usually imperceptible to human observers without amplification. Among teleosts, the separate ability to produce “strong” electric discharges for the purposes of prey capture or defense against predators has arisen once in the gymnotiform *E. electricus* (known commonly as the electric eel), not at all in the African mormyroids, and once in the African electric catfish family Malapteruridae. While *E. electricus* is additionally capable of active electroreception using weak electric discharges, as are all other gymnotiforms, electric catfishes are not. Hereafter, for simplification, South American weakly electric fishes refer to all gymnotiforms including *E. electricus*.

**Figure 2:**
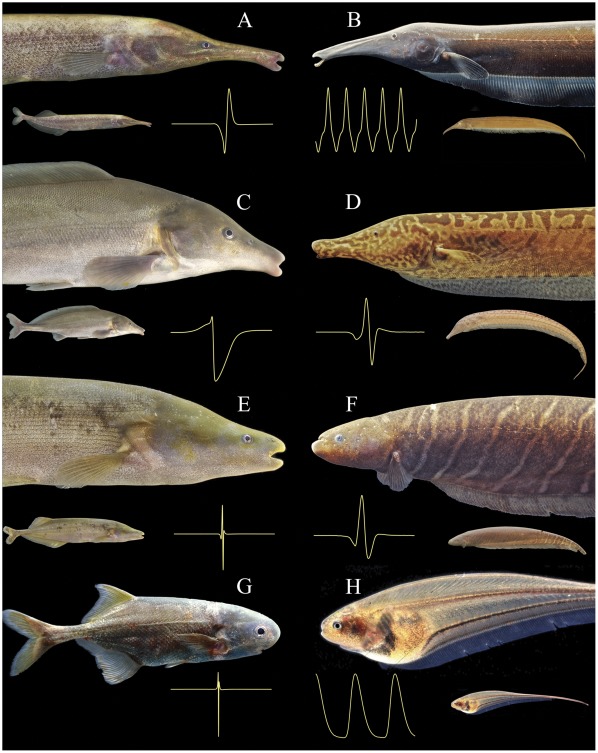
Morphological convergences between African and South American electric fishes. Mormyroid African electric fishes (left column) are facing gymnotiform South American electric fishes (right column) with similar aspects of morphology (such as elongate bodies, extended tube-like snouts, reduced eyes, and/or small mouth sizes). Anterior portion of body shown above small image of whole body (except for *Petrocephalus sullivani*); electric organ discharge waveform shown for every species (each trace 5 ms in total duration with head-positivity plotted upwards). (**A**) *Mormyrops zanclirostris*, 175 mm standard length (SL), Ivindo River, Gabon, (**B**) *Sternarchorhynchus oxyrhynchus*, 220 mm total length (TL), Rio Negro, Brazil; (**C**) *Mormyrus proboscirostris*, 232 mm standard length, Ubundu, Congo River, D.R. Congo; (**D**) *Rhamphichthys* sp., 305 mm TL, Rio Negro, Brazil; (**E**) *Mormyrops anguilloides*, 195 mm SL, Yangambi, Congo River, D.R. Congo; (**F**) *Gymnotus* sp., 195 mm TL, Rio Negro, Brazil; and (**G**) *Petrocephalus sullivani*, Ogooué River, Gabon; (**H**) *Eigenmannia* sp., Apure River, Venezuela. Species A–D feed on benthic invertebrates, species E, F are piscivorous, and G, H feed on pelagic invertebrates.

Arising from dissimilar, non-electrogenic teleost ancestors, mormyroid and gymnotiform fishes are phenotypically similar in a number of ways. First and foremost is the general presence of electrogenesis and electroreception. Additionally, mormyroids and gymnotiforms exhibit striking convergence in specific aspects of their body form, swimming behavior, reproductive behavior, ecology, nocturnal activity patterns, electric signals, and even the neuronal algorithms used to avoid jamming of active electrolocation and communication [9], [33]–[41]. Some examples of convergence in body form are illustrated in Fig. 2.

Convergent evolution may be defined as the independent evolution of similar biological traits from dissimilar ancestral states in unrelated lineages. In addition to convergent evolution, weakly electric fishes can also be characterized by parallel evolution; the latter term is often used to describe independent but similar patterns of trait divergence from the same ancestral trait, regardless of whether the parallel pattern of divergence occurred in closely or distantly related lineages.

From the perspective of evolutionary developmental biology, gymnotiforms and mormyroids exhibit extraordinary evolutionary parallelism in at least two important ways. First, both gymnotiforms and mormyroids evolved novel myogenic electric organs (EO_myo_) that are developmentally derived from skeletal muscle progenitor cells (myoblasts) [42]–[47]. Further, at genetic and molecular levels, Zakon et al. [20] and Arnegard et al. [19] demonstrated, in both groups, that the same sodium channel α-subunit paralog (i.e. gene duplicate) was co-opted from skeletal muscle for exclusive expression in EO_myo_, and that similar patterns of amino acid substitution subsequently occurred in regions of the α-subunit thought to contribute to electric signal variation. Second, both groups are characterized by the origin of high frequency electroreceptors (underlying active electrolocation and electrocommunication), which are derived from similar lateral line receptor precursors [30], [48]–[52]. The parallel origins of these complex traits on both the sender and receiver sides of electrical signaling offers an opportunity to investigate generalized patterns (or “rules”) underlying the origins of evolutionary novelty, a topic of great current interest [53]–[55]. While acknowledging that weakly electric fishes are characterized by both convergent and parallel evolution, depending on one’s perspective, we refer to these fishes hereafter as exemplifying a textbook example of convergent evolution [56]–[58]; we do so in order to highlight the independent origins of Mormyroids and Gymnotiforms from phylogenetically unrelated and phenotypically dissimilar teleost ancestors.

Previously, Lissmann [58] proposed that convergence upon an anguilliform body form with ribbon fin propulsion, present in all gymnotiforms and some mormyroids, might have been a way to minimize bending of the body axis during active electrolocation. He reasoned that such adaptations would have been an advantage in the early evolution of active electrolocation because they would reduce the amplitude modulations in the local electric fields that might have confounded the detection of objects in the environment. Attractive as the rigid body hypothesis was at the time, recent work suggests that it may not be that important for modern extant species, which have convergently evolved cerebellum-like neural circuitry in the hindbrain capable of learning, to cancel the amplitude modulations of the electric organ discharge caused by tail movements [59]–[65]. Others have suggested that ribbon-fin propulsion may provide enhanced maneuverability with reduced turbulence when electrolocating and approaching prey organisms [66].

There is even more reason to believe that other aspects of convergence in body form between mormyroids and gymnotiforms result from the selection pressures imposed by their shared electrosensory and electrocommunication systems. For example, Heiligenberg [67] suggested that the elongate body form with the electric organ located far from the head and trunk might be an adaptation for extending the effective distance of active electrolocation. Similarly, Hopkins [68] suggested that extending the length of the tail with its electric organ may have been a way of increasing the voltage of the electric organ discharge and hence the active space of electric signaling, which would have been important especially in water with reduced conductivity. Stoddard [69] proposed additional adaptations involving electrogenesis and electroreception for avoiding predation by electroreceptive species. Some of the striking adaptations seen in body form, such as the elongate snouts or “trunks” of some mormyroids and gymnotiforms (e.g. Fig. 2), may have arisen secondarily, long after the origins of active electrolocation, as a consequence of the types and habits of prey organisms most readily acquired in the novel ecological niches exploited by active electrosensory predators. Other convergent evolutionary shifts, such as reduced mouth sizes and restricted gill openings (Fig. 2), likely result from the need for reducing interfering electrical emissions from electrically active epithelial tissues. Additionally, the small eye sizes of many, but certainly not all, mormyroids and gymnotiforms may be a direct result of their active electrosensory systems superseding the “passive” sense of vision in importance (Fig. 2). The convergence of electric signals on either wave discharges or pulse discharges (e.g. see Fig. S1 of Arnegard et al. [19]) has stimulated much discussion in the literature, but there is no general consensus on the causes of these remarkable cases of signal convergence.

### Prior Work Attempting to Date the Convergent Origins of Mormyroids and Gymnotiforms

Uncertainty surrounding the dates of origin of the Gymnotiformes and the Mormyroidea, and the timing of important innovations in electrolocation and electrocommunication, is a function of the poor fossil record of weakly electric fishes. The most ancient gymnotiform fossil excavated to date is †*Humboldtichthys kirschbaumi* from the Upper Miocene (ca. 8–10 millions of years ago [Mya]) of Bolivia [70]–[71]. The fossil record of the Mormyroidea is known only by some teeth of †*Gymnarchus* sp. recently described from the late Eocene (37 Mya) of Egypt [72] and some remains of †*Hyperopisus* sp. from the Plio-Pleistocene (i.e., more recent than 5.3 Mya) of Lake Edward and the Semliki River in Congo [73]–[75]. Hence, early hypotheses for the ages of the Gymnotiformes and Mormyroidea were based on phylogenetic and biogeographical hypotheses made for the large and relatively fossil-rich groups of teleosts to which they belong: the Ostariophysi and Osteoglossomorpha, respectively. Studies based on paleontological and biogeographical considerations have achieved little consensus on the ages of origin and diversification of these two groups of electric fishes, with some placing these events before [76]–[78] and others after [79], [80] the complete separation of Africa and South America, dated to the period from ca. 110 to 100 Mya [81].

The first molecular efforts aimed at estimating ages of the Gymnotiformes and Mormyroidea were based on short sequences of nucleotides (<850 bp) or amino acids (<750 positions), with sparse taxonomic sampling and the use of strict molecular clocks calibrated with rates derived from other groups of fishes and/or from fossils and geological events [82], [83] (also see Table 1). In these studies, ages of the Mormyroidea and Gymnotiformes were separately estimated using distinct sets of taxa and/or character data. Alves-Gomes [82] and Kumazawa and Nishida [83] estimated the age of the stem Mormyroidea to 61–72 Mya and to 241+/−23 Mya, respectively; the age of the Gymnotiformes was estimated to 79–117 Mya by Alves-Gomes [82]. The introduction of maximum likelihood and Bayesian methods to reconstruct the divergence times using complete mitochondrial sequences yielded revised estimates for the ages of these groups (Table 1). Nevertheless, these studies still included few electric fish species/lineages, with poor coverage of the morphological and taxonomic breadths of these groups. Peng et al. [84] proposed that the crown group Gymnotiformes is 150 My old and that the gymnotiform lineage originated 197 Mya, whereas Nakatani et al. [85] pushed back the origin of the crown and stem group Gymnotiformes to 189 Mya and 226 Mya, respectively. Inoue et al. [86] estimated the age of the crown group Mormyroidea to 142 Mya and the age of the mormyroid lineage to 162 Mya. Lavoué et al. [87] estimated the age of the crown group Mormyroidea to 85.2 Mya or 136.0 Mya, depending on the calibration method considered, and the origin of the stem group Mormyroidea to 104.2 Mya or 159.7 Mya.

**Table 1:** Previously estimated ages of the Mormyroidea and the Gymnotiformes.

	Age of the crown-group Mormyroidea	Age of the stem-group Mormyroidea	Age of the crown-group Gymnotiformes	Age of the stem-group Gymnotiformes
**Fossil record**:	**37 Mya**, †*Gymnarchus* sp.; Murray et al. [72]	**93.6–99.6 Mya**, †*Palaeonotopterus*; Foreyet al. [148]	**ca. 8–10 Mya**, †*Humboldtichthys*; Gayetand Meunier [70]	**83.5–88.6 Mya**, earliest catfish fossil; Patterson [94]
**Alves-Gomes** [**82**] (Partial 12 Sand 16 S rRNAs, strict molecular clock, fossil-based calibrations)	**-**	**60.7–72.0 Mya** (date for divergence of the Mormyroidea and the Notopteridae)	**–**	**79.4–117.6 Mya** (date for divergence of the Gymnotiformes and the Siluriformes)
**Kumazawa and Nishida ** [**83**] (ND2 and cytochrome *b*, strict molecular clock, fossil- and geological-based calibrations)	**79±12 Mya** (date for divergence of *Brienomyrus niger* 1 and *Campylomormyrus* sp.)	**242±23 Mya** (date for divergence of the Mormyroidea and the Osteoglossidae)	**–**	**–**
**Peng et al. ** [**84**] (Mitogenomes,relaxed molecular clock, fossil-based calibrations)	**–**	**–**	**150 Mya** [95% CI not specified] (date fordivergence of *Eigenmannia*and *Apteronotus*)	**197 Mya** [95% CI not specified] (date for divergence of the Gymnotiformes and the Characiformes^2^)
**Inoue et al. ** [**86**] (Mitogenomes, relaxed molecular clock, fossil-based calibrations)	**142 Mya** [95% CI = 120−165 Mya] (date for divergence of *Gymnarchus*and the Mormyridae)	**162 Mya** [95% CI = 138−186 Mya] (date for divergence of the Mormyroidea and the Notopteridae)	**–**	**–**
**Nakatani et al. ** [**85**] (Mitogenomes, relaxed molecular clock, fossil-based calibrations)	**–**	**–**	**189 Mya** [95% CI = 166−212 Mya] (date forthe stem group of theGymnotidae)	**226 Mya** [95% CI = 206−245 Mya] (date for the crown group of the Characiphysae)
**Lavoué et al. ** [**87**] (Mitogenomes, relaxed molecular clock, two fossil-based calibration methods: their reconstructions #1 and #2)	**their reconstruction #1: 85.3 Mya** [95% CI = 55.7−111.8 Mya]; **their reconstruction #2:** **136.0 Mya** [95% CI = 101.7−173.7 Mya] (both dates are for the divergenceof *Gymnarchus* and the Mormyridae)	**their reconstruction #1:** **104.2 Mya** [95% CI = 77.9−125.6 Mya]; **their reconstruction #2:** **159.7 Mya** [95% CI = 121.6−197.0 Mya] (both dates are for the divergence of the Mormyroidea and the Notopteridae)	**–**	**–**

1 Identified as *Marcusenius* sp. in Kumazawa and Nishida [83]; ^2^the order Characiformes was found not to be monophyletic by Peng et al. [84].

Ages were inferred from direct evidence (based on the fossil record, with strict minimum ages [70], [72], [94], [148]) or via indirect evidence (molecular-based estimates [82]–[87]). Ages are given as millions of years ago (Mya). Following convention, daggers (†) indicate extinct taxa.

None of these recent studies has employed broad enough taxonomic sampling and a sufficiently large molecular dataset to simultaneously and robustly estimate ages of the Gymnotiformes and the Mormyroidea from a single tree. Here, we re-examine the ages of these two groups using: (1) complete mitochondrial genomes as our character set; (2) a unique and extensive taxonomic sampling including several basal teleost species and 27 species of Mormyroidea and Gymnotiformes, representing all families of weakly electric teleosts; and (3) a relaxed-clock Bayesian method that infers phylogenetic relationships and divergence times simultaneously, given constraints that are enforced on the basis of multiple fossil calibration points. We go on to discuss the significance of the resulting timeframe for investigating the origins of evolutionary novelty, as well as the influence of innovations in communication on species radiation, using the unified weakly electric fish system.

## Results

### Phylogenetic Relationships

**Figure 3:**
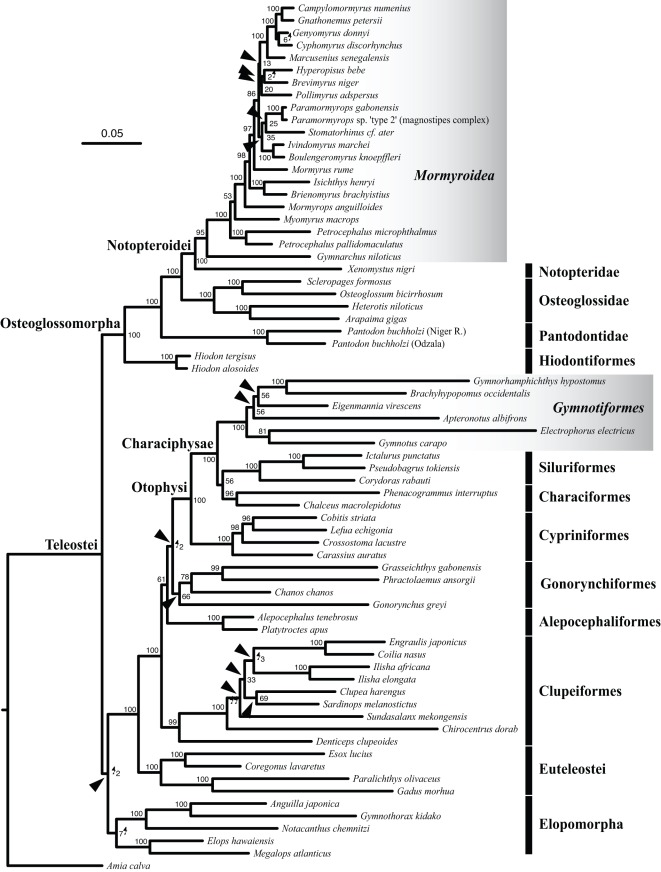
Best maximum likelihood tree of the Teleostei from analysis of the *mt-seq* data subset “12RT,” using the software RAxML. Branch lengths are proportional to the number of substitutions per nucleotide position (scale bar  = 0.05 substitutions). Numbers at nodes give node support in terms of bootstrap proportions. The tree is rooted with *Amia calva*. Light grey gradient boxes highlight the Mormyroidea (African weakly electric fishes) and the Gymnotiformes (South American weakly electric fishes). Arrowheads indicate nodes for which topological differences were found compared to trees reconstructed using the two other data subsets (“123ryRT” and “123RT,” shown in Fig. S1 and S2, respectively).

Maximum likelihood (ML) analyses of the different data subsets yielded similar phylogenetic results, with topological differences only occurring for a few of the relationships (Fig. 3, S1, and S2). In all ML analyses across all three data subsets, the Gymnotiformes and the Mormyroidea are each monophyletic to the exclusion of all other teleosts (bootstrap proportion [BP]  = 100%). Moreover, these two clades of weakly electric fishes are nested within two distantly related groups of Teleostei, the Ostariophysi and the Osteoglossomorpha. Because the Osteoglossomorpha (with or without the Elopomorpha) is the most basal lineage in our tree, the most recent common ancestor of the Ostariophysi and the Osteoglossomorpha is also the most recent common ancestor of the crown-group Teleostei (Fig. 3).

Confirming a number of other studies [32], [80], [86]–[89], we found that the sister group of the Mormyroidea within the Osteoglossomorpha is the family Notopteridae (BP  = 100%). In contrast to an established morphological hypothesis [28], however, we inferred that the sister group of the Gymnotiformes within the Ostariophysi is the clade (Characiformes, Siluriformes). Although this relationship appears stable in our results, it is only moderately supported by BP (from 47% to 96%).

Higher-level relationships within the Mormyroidea also agreed with previous studies: we found *Gymnarchus niloticus* (i.e., the Gymnarchidae) to be the sister taxon of the Mormyridae [19], [24], [90], [91] (BP  = 100%); and we found the family Mormyridae to be divided into two lineages, the Petrocephalinae (*Petrocephalus*) and the Mormyrinae (all remaining genera) [24], [90], [91] (BP = 100%). Within the Mormyrinae, *Myomyrus* appears to be the most basal lineage followed by the genus *Mormyrops*. These nested relationships are fully consistent with the findings of Sullivan et al. [24], Lavoué et al. [22], and Arnegard et al. [19]. The clade (*Isichthys*, *Brienomyrus*) and the genus *Mormyrus* do not form a monophyletic group containing these three genera as found by Lavoué et al. [22]; instead, they are sequential sister groups of all remaining mormyrins [24]. Phylogenetic relationships among the rest of the Mormyrinae in the trees are not better supported by our results than by earlier molecular studies [19], [22], [24]. We found some of these relationships to be inconsistent from one analysis to the other in the present study, and with respect to the above-mentioned molecular studies.

Across our different phylogenetic analyses, we found topological instability among the four gymnotiform lineages represented in our taxonomically limited dataset: (*Gymnotus*, *Electrophorus*); *Eigenmannia*; (*Gymnorhamphichthys*, *Brachyhypopomus*); and *Apteronotus*. According to data subset “12RT” (Fig. 3), the clade (*Gymnotus*, *Electrophorus*) appears to be the sister group to the remaining gymnotiform taxa, successively followed by *Apteronotus*, *Eigenmannia*, and the clade (*Gymnorhamphichthys*, *Brachyhypopomus*). By contrast, according to the two other data subsets (“123ryRT” and “123RT,” Fig. S1 and S2 respectively), *Apteronotus* appears to be the most basal group, followed by either the clade (*Gymnotus*, *Electrophorus*) or *Eigenmannia*. The topology from our data subset “123ryRT” is fully congruent with a gymnotiform phylogeny recently inferred by Arnegard et al [19], which they found to be robust across two independently evolving nuclear genes. When we constrained our two other topologies to that of Arnegard et al. [19], we found that the best likelihood score of the constrained trees did not differ statistically from that of our best-unconstrained tree (based on AU test results; data not shown). Thus, the general findings from our *mt-seq* data are unable to reject the phylogenetic hypothesis of Arnegard et al. [19].

Importantly, nodes representing the two independent origins of electrogenesis in weakly electric teleosts, the dating of which was the main aim of our study, received extremely strong topological support under all data subsets and analyses.

### Divergence Time Estimation

**Figure 4:**
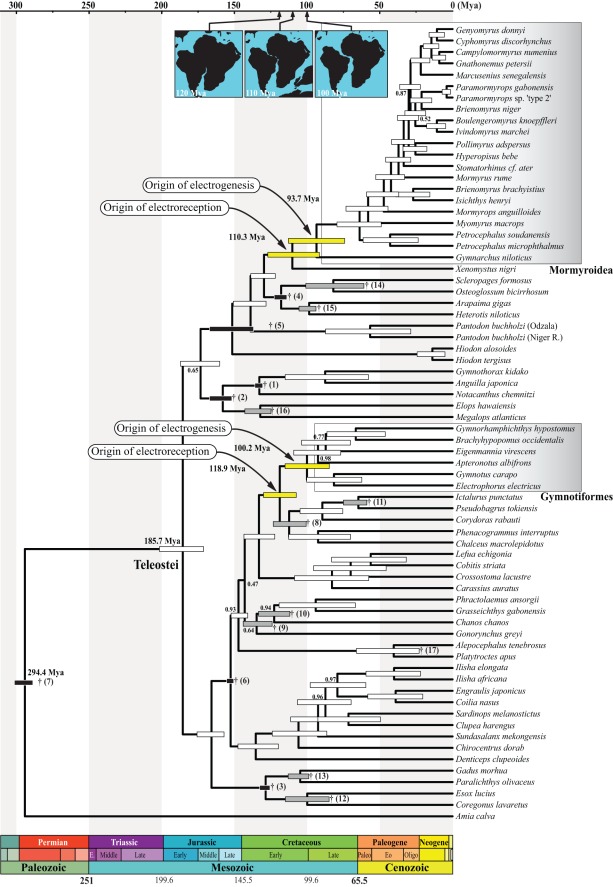
Phylogenetic chronogram of the Teleostei based on a Bayesian relaxed clock approach using the *mt-seq* data subset “12RT” under the first fossil calibration strategy. In this approach, we used seven fossil-derived calibration constraints following lognormal distributions and ten others following uniform distributions (i.e., reconstruction #1). *Amia calva* is used to root the tree. Light grey gradient boxes highlight the Mormyroidea and Gymnotiformes. Horizontal timescale is in millions of years ago (Mya). Only selected epoch names are given. Abbreviations: E, early; Paleo, Paleocene; Eo, Eocene; and Oligo, Oligocene. Standardized timescale colors taken from the Commission for the Geological Map of the World. 95% age credibility intervals are shown as black and grey horizontal bars (calibration constraints on corresponding nodes), yellow horizontal bars (focal nodes of interest), and white horizontal bars (all other nodes). Daggers indicate that minimum ages were used to calibrate the nodes, and adjacent numbers in brackets refer to source fossils listed in the Materials and Methods. Numbers at nodes are the posterior probability support values (shown only when <1). Timing of the separation of Africa and South America is depicted by the three insets at the top, modified from [81]. Here, “origin of electroreception” refers to the initial origin of any kind of electroreceptive system in the broadest sense (see text for elaboration).

**Figure 5:**
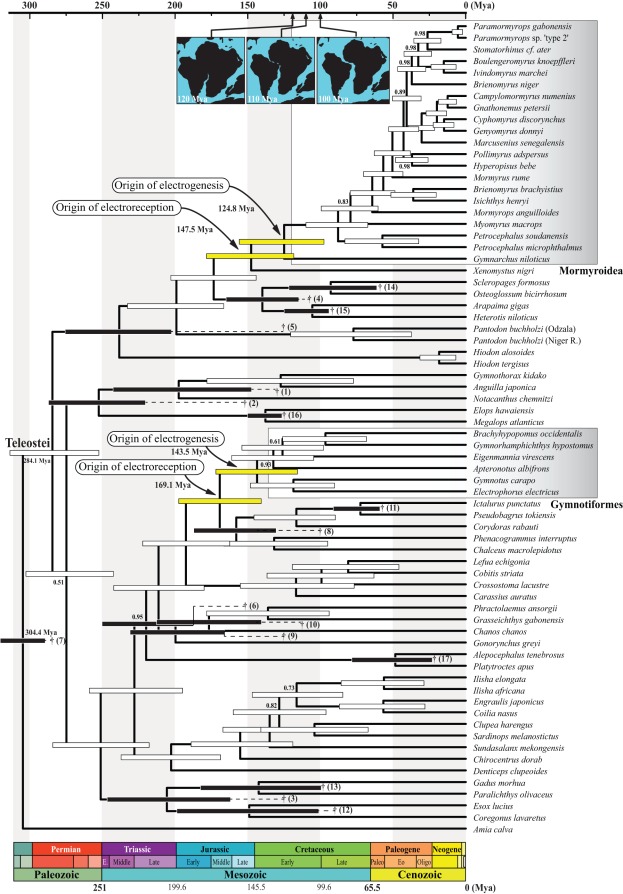
Phylogenetic chronogram of the Teleostei based on a Bayesian relaxed clock approach using the *mt-seq* data subset “12RT” under the second fossil calibration strategy. In this approach, we used 17 fossil-derived calibration constraints following uniform distributions (i.e., reconstruction #2). 95% age credibility intervals are shown as black horizontal bars (calibration constraints on corresponding nodes), yellow horizontal bars (focal nodes of interest), and white horizontal bars (all other nodes). Daggers indicate that minimum ages were used to calibrate the nodes, and adjacent numbers in brackets refer to source fossils listed in the Materials and Methods. Dashed lines between daggers and lower age limits of corresponding nodes (within the 95% age credibility intervals) depict putative ghost lineages in the fossil record. All other details as in Fig. 4.

The two methods used to calibrate our chronogram produced different age estimates for the independent origins of weakly electric teleost fishes, as well as for deeper nodes in the tree (Fig. 4 and 5). Using reconstruction #1 (with strong maximum age constraints, Fig. 4), the age of the crown-group Notopteroidei and the age of the crown-group Mormyroidea were estimated to be 110.3 Mya (95% credibility interval [CI]  = 91.7–127.2 Mya) and 93.7 Mya (CI  = 74.3–112.9 Mya), respectively. The age of the most recent common ancestor of the clade (Gymnotiformes, (Siluriformes, Characiformes)), named the Characiphysae by Wiley and Johnson [92], was estimated to be 118.9 Mya (CI  = 107.6–130.1 Mya), and the age of the crown-group Gymnotiformes was estimated to be 100.2 Mya (CI  = 84.9–115.3 Mya). The second method of reconstruction (#2, with only soft maximum age constraints, Fig. 5) yielded uniformly older age estimates for the entire tree, with the ages of the Notopteroidei, the crown-group Mormyroidea, the Characiphysae, and the crown-group Gymnotiformes estimated to 147.5 Mya (CI  = 117.9–177.9 Mya), 124.8 Mya (CI  = 97.5–155.7 Mya), 169.1 Mya (CI  = 140.5–197.3 Mya), and 143.5 Mya (CI  = 115.8–171.8 Mya), respectively.

**Figure 6:**
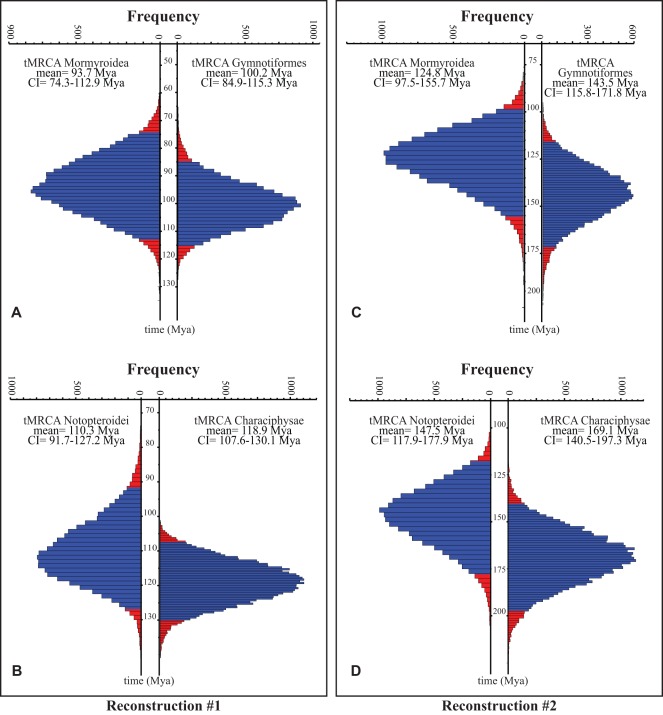
Distributions of estimated ages for focal nodes of interest under each fossil calibration scheme. For each plot, estimated ages were sampled every 5,000 generations from two independent BEAST runs of 1×10^8^ generations each. Resulting age histograms are shown for the estimated times of the most recent common ancestors (tMRCAs) of the Mormyroidea and the Gymnotiformes (**A**) under reconstruction #1 (also see Fig. 4) and (**C**) under reconstruction #2 (also see Fig. 5); histograms are similarly shown for tMRCAs of the Notopteroidei and the Characiphysae under (**B**) reconstruction #1 and (**D**) reconstruction #2. The span of blue bars along the vertical axis of each plot gives the 95% credibility interval for that particular age estimate. Tails of each distribution are shown in red. All time scales in millions of years ago (Mya).

With each reconstruction method, the mean ages of the Mormyroidea and the Gymnotiformes were found to be very similar to each other (<15% difference under both calibration strategies), with largely overlapping credibility intervals (Fig. 6). The mean ages of the Notopteroidei and Characiphysae were also quite similar between methods, with the Characiphysae slightly older than the Notopteroidei (Fig. 6). Nodes defining the latter two clades also define minimum age estimates for the independent origins of electroreception among teleosts (i.e., in the form of derived ampullary electroreceptors; see Fig. 1). Strikingly, the intervals between these estimates for the early origins of teleost electroreception, in this broadest sense of any form of electroreception, and the corresponding estimates for the origins of electrogenesis are quite similar for the two lineages of weakly electric fishes: the mean estimated interval is 16.6 My for mormyroids vs. 18.7 My for gymnotiforms under reconstruction #1, and 22.7 My vs. 25.6 My under reconstruction #2). Thus, the independent origins of electrogenesis may have occurred after roughly the same intervals of time following the independent origins of teleost electroreception in the broad sense. However, these nodes define only minimum ages for the origin of electroreception and electrogenesis, respectively. Each trait actually evolved somewhere along the stems that subtend those nodes, and we cannot estimate the actual origin of these novel innovations with any greater precision.

Despite these points of uncertainty, our results provide evidence for comparable and perhaps nearly simultaneous dates for the origins of African and South American weakly electric fishes. We show that the origins of these groups occurred well after the splitting of the respective lineages from their most recent common ancestor, which we date to 185.7 Mya (CI  = 171.4–201.7 Mya) under reconstruction #1 or 284.1 Mya (CI  = 252.2–313.3 Mya) under reconstruction #2.

## Discussion

### Dating the Two Independent Origins of Electrogenesis in Teleosts

In the present study, we have made the first effort to simultaneously infer ages for the South American Gymnotiformes and the African Mormyroidea by analyzing a single, coherent molecular dataset that includes multiple representatives of both groups, as well as each of their possible sister groups. Using whole mitochondrial genome sequences and fossil calibration points for several nodes within teleosts, we made the surprising finding that gymnotiforms and mormyroids originated independently from non-electrogenic ancestors at about the same period of time in the Earth’s history. The close occurrences of these events in time appear robust across our two methods of fossil-based calibration, although the absolute date estimates differ somewhat between the two methods. Under reconstruction #1, crown-group gymnotiforms and mormyroids are estimated to have originated 100.2 and 93.7 Mya, respectively; under reconstruction #2, the respective estimates are 143.5 and 124.8 Mya.

Constraining the trees with seven maximum ages equal to the upper age limits of the strata from which fossils were excavated (reconstruction #1) provided conservative age estimates. Not surprisingly, we found such estimates to be roughly congruent with the general temporal framework of teleost diversification established by paleoichthyologists (e.g. [93], [94]). Much older divergence time estimates were obtained when we relaxed all maximum age constraints based on our selected fossils (reconstruction #2). Under this second method of calibration, for example, the estimated origin of the crown-group Teleostei was pushed back before the Mesozoic Era, to around 284 Mya. This estimate predates the first crown group teleost fossil by more than 130 My but is largely in agreement with several other molecular dating studies of the Teleostei with similarly soft maximum age constraints [84]–[86], [95], [96].

At present, we have no robust means of deciding which of our divergence time reconstructions provides the best estimates for the absolute ages of the Gymnotiformes and the Mormyroidea. However, our use of two very different calibration methods brackets a reasonable range of times for them. Further studies aiming to investigate divergence times within the teleosts will likely help to refine this range. Most importantly, regardless of the method of fossil-derived calibration (and, therefore, the reconstructed timeframe of divergence), the estimated mean dates for the origins of South American and African weakly electric fishes are very similar, with largely overlapping distributions (Fig. 6).

### Electrogenesis in Teleosts: Two Late by-products of the Teleostean Whole Genome Duplication

Gene duplication is thought to be an important source of raw material for the origin of novel traits [97]–[100]. Recent investigation of a duplicated voltage-gated sodium channel gene (*Scn4aa*) in electric fishes has suggested that whole genome duplication (WGD) just prior to the radiation of teleosts [95], [101], [102] contributed to the origin of novel electrogenic systems in mormyroids and gymnotiforms [19], [20], [103]. The chronogram generated from reconstruction #1 suggests that the WGD event occurred at least 85.5 My before the two independent origins of weakly electrogenic fishes (at least 140.6 My under reconstruction #2). Thus, as discussed by Arnegard et al. [19], gene duplication appears to be capable of making important contributions to parallel origins of novelty even when gene duplicates are co-opted for innovation long after the duplication event occurs.

### Significance of the Similar Timing Estimated for the Independent Evolution of Weakly Electric Fishes

Myogenic electric organs possessed by mormyroids and gymnotiforms are remarkably similar in many ways, though there are also important differences between lineages and great diversity in structure and function among species [40], [42]–[44], [104]. By demonstrating that the two groups of weakly electric fishes originated at nearly the same time in evolutionary history, we show that very similar amounts of time passed between the WGD at the base of the teleost radiation and the origins of novel myogenic electric organs within each of these lineages. Moreover, in each of the two independent groups of weakly electric fishes, we are peering back through roughly the same amounts of time to the origins of complex novel communication systems, as well as the effects of these key innovations [13], [19] on the subsequent radiations of sub-lineages and species. Our finding of these temporal similarities adds to many other lines of evidence, which we summarize in the Introduction, suggesting that weakly electric fishes present one of the most impressive, and potentially empirically valuable, cases of convergent evolution among vertebrates.

Perhaps as significant as the contemporaneous origins of mormyroids and gymnotiforms is our striking finding that very similar periods of time may have elapsed between the origin of passive electroreception and the appearance of the myogenic electric organ (and active electroreception) in each of the two lineages of weakly electric fishes. Even when one reconstructs the origin of a trait as occurring along a particular branch in a phylogeny (using the principle of parsimony, for example), there still remains much uncertainty as to when that trait actually arose along the identified branch. While acknowledging this uncertainty, we also note that the convergent evolution of myogenic electric organs and similar electrical communication systems in two distantly related teleost groups essentially offers a rare case of a single, albeit rough, degree of freedom left over for evaluating generalizations about evolutionary processes. In this context, the similarity of intervals between the appearance of any form of electroreception, initially associated with ampullary electroreceptors tuned to low electrical frequencies, and the subsequent origin of myogenic electric organs deserves some note. Our best estimates for these intervals fall within 16–19 My or 22–26 My under reconstructions #1 and #2, respectively. We assume that selection pressures for weak electric organs could only have arisen sometime after an electrosensory system began to evolve, as electroreception is required for the detection of reafferent signals produced by electric organs. Under this assumption, the above intervals provide rough upper estimates for the time period required for evolution to construct a weak, myogenic electric organ *de novo* from its skeletal muscle precursor.

Both mormyroids and gymnotiforms have diversified on their respective continents to a similar degree, each group comprising close to 200 named species [27]. Using comparative methods, investigators recently inferred the positive influences that a novel communication modality (in this case, electrical communication in mormyroids) and its component neural traits can exert on the opportunity for signal evolution by increasing the number of axes of variation in signal space [13], [16], [17]. They further showed that this may have the effect of augmenting the rate of signal divergence, and thus, the tempo of species radiation. Similar comparative studies have not yet been attempted within the Gymnotiformes, though they will likely be very enlightening. When such complimentary investigations are attempted, an established timeframe–presently indicating very similar periods over which these distantly related groups have radiated–will help to further inform comparisons between mormyroids and gymnotiforms.

### Geographical Origin and Early Biogeography of the Gymnotiformes and Mormyroidea

The historical biogeography of the Osteoglossomorpha and the Ostariophysi has been extensively examined in relation to the tectonic history of the Earth and the fragmentation of the supercontinent Gondwana [76]–[78], [85], [105]–[108]. Both groups are good candidates for such biogeographic studies: they are predominantly restricted to freshwater, having an intolerance to salinity (yet, several marine fossils in both groups have been discovered); both groups also exhibit worldwide intercontinental distributions, and their respective fossil records indicate ancient origins predating the fragmentation of Gondwana. However, the absence of gymnotiform and mormyroid fossils older than 35 My has limited the inferences that can be made from the fossil record about their geographical origins and early diversification.

Tectonic map reconstructions show that the splitting of Africa and South America started at about 130 Mya. These continents were fully disconnected at about 100 Mya, by which time a complete north-south marine channel was fully established [81], [109]–[111]. Eustatic sea level reconstructions during the period from 130 Mya to 100 Mya indicate that the level of the Earth’s oceans was on average 100 meters higher than it is today [112], [113], which likely accentuated the separation between continents. Freshwater drainages on each continent appear to have been separated by a permanent seaway by around 110 Mya, which is congruent with the observed distribution of freshwater and marine fossils at this time [114], [115]. Our two reconstructions (i.e., estimates) for the origin of the Gymnotiformes and the Mormyroidea straddle this date of 110 Mya for the complete separation of Africa and South America. Reconstruction #1 favors a post-separation origin for both crown groups (100.2 Mya and 93.7 Mya, respectively), whereas reconstruction #2 infers the ages of the gymnotiform and mormyroid crown groups to be 143.5 Mya and 124.8 Mya, respectively, corresponding to the period before the complete separation of Africa and South America. Conclusively demonstrating the origin of these groups before the final breakup of Gondwanaland, however, will require the discovery of gymnotiform or mormyroid fossils older than 110 Mya.

Our divergence time reconstructions thus illustrate the long and independent histories of both electric fish groups on each continent: the origin and diversification of crown group Gymnotiformes and Mormyroidea began at the dawn of the independent histories of South America and Africa in the early Late Cretaceous. These analyses suggest the possibility that the absence of living or fossil gymnotiforms from Africa, and of mormyroids from South America, may simply be a consequence of their time of origin: these lineages may have never co-occurred on western Gondwana, or some portion of it, before it was functionally divided.

### Sister Groups of the Gymnotiformes and the Mormyroidea: Dating the Origins of Electroreception

Fink and Fink [116], in their morphological comparative study of the Ostariophysi (i.e., Otophysi plus Gonorynchiformes), proposed that the Gymnotiformes and the Siluriformes form a monophyletic group based on 22 morphological synapomorphies (shared, derived traits). In addition, Fink and Fink [116] noted that the Siluriformes and the Gymnotiformes are also the only electroreceptive fishes within the Ostariophysi: the Characiformes, Cypriniformes, and Gonorynchiformes lack any form of electroreception. The work of Fink and Fink [116] and its revised version [28] has been a cornerstone of the systematics of these fishes: it represents the first and only comprehensive cladistic study of the Ostariophysi. Only seven of the 127 morphological characters listed in Fink and Fink [116] are homoplasious, yielding an extraordinarily well-supported phylogenetic hypothesis. Despite this, our result points toward the possibility of an alternative hypothesis, with moderate but consistent support for the Gymnotiformes being the sister group to the clade (Siluriformes, Characiformes), and not to the Siluriformes alone.

While previous molecular studies have provided strong support for the Characiphysae, the clade consisting of characiforms, gymnotiforms, and siluriforms (C, G, S), none has ever recovered the Fink and Fink hypothesis of ((G, S) C). Several have reported ((S, C) G) as we do here [85], [117]–[122], or ((G, C) S) [123]–[125], or they have been mostly inconclusive [126], [127]. Phylogenetic investigation of many more taxa from the Siluriformes and the Characiformes is needed to better understand the nature of this incongruence.

Importantly, the ((S, C) G) topology does not require a change to the hypothesis that an ampullary electroreceptor-based system originated only once in the Characiphysae, although it does requires the additional step of a loss of the ampullary electroreceptors subsequently in the characiform lineage. This hypothetical situation would be similar to the one favored for the Notopteroidei, where the Asian notopterids are thought to have lost electroreception [32].

### Conclusion

The origins of the South American and African weakly electric fishes have been challenging to evaluate due to the incompleteness of the fossil record and the lack of robust molecular dating methods applied to a comprehensive molecular dataset consisting of all relevant taxa. Our study provides such molecular evidence for the phylogenetically independent, but more or less contemporaneous, origins of the Gymnotiformes and Mormyroidea, at least many tens of millions of years after their most recent common ancestor lived. Moreover, our findings suggest that a similar amount of time elapsed in each group between (1) the initial origin of any form of electroreception in these lineages and (2) the subsequent origin of electrogenesis, which was accompanied by the further evolution of more complex electroreceptive systems [16]. We also found that the Gymnotiformes and the Mormyroidea arose around the time of the final fragmentation of western Gondwana, but we cannot specify whether these events occurred before or after the complete separation of Africa and South America. Given the temporally similar, yet phylogenetically independent origins of the two electrogenic groups of teleosts, it is tempting to speculate that some shared environmental condition during the early Late Cretaceous, such as the climate, may have contributed to their contemporaneous origins. While it is not possible to rigorously evaluate such an hypothesis, placing the origin and diversification of African and South American electric fishes near the beginning of the separate histories of their respective continents adds a new perspective to this extraordinary, and scientifically valuable, example of convergent evolution in vertebrates.

## Materials and Methods

### Terminology

“Stem group” and “crown group” are useful terms when discussing the origin (stem-) and diversification (crown-) of an extant group of organisms. A crown group includes the most recent common ancestor of a living monophyletic group plus all of its descendants, living or extinct [128]. In our terminology here, a stem group includes the most ancient common ancestor of a living monophyletic group plus all of its living and extinct descendants. In other words, a stem group includes all the descendants of an extant lineage that arose anytime after the split with its living sister lineage.

### Taxon Sampling Strategy and Mitogenome Sequencing

We designed the taxonomic sampling strategy for our study to simultaneously estimate the relative ages of the stem- and crown-group gymnotiforms and mormyroids. This required the inclusion of their putative sister groups, the Siluriformes and the Notopteridae respectively, as well as a large sampling of so-called basal teleost fishes. Within the Gymnotiformes, we selected one representative from all but one of the constituent families (Sternopygidae, Apteronotidae, Gymnotidae, Hypopomidae, and Rhamphichthyidae) and two representatives from the remaining family (Gymnotidae). Within the Mormyroidea, we included *Gymnarchus niloticus* (Gymnarchidae) plus 19 representatives of the Mormyridae representing each major lineage [22], [24].

Seventeen complete, or nearly complete, mtDNA sequences (hereafter “*mt-seqs*”) of African and South American electric fishes were newly obtained for this study. In only a few cases, a short portion of the control region was undetermined due to long thymine repeats. We combined our new data with the following previously published sequences: *mt-seqs* for four African electric fishes (*Gnathonemus petersii*, *Myomyrus macrops*, *Petrocephalus microphthalmus*, and *Petrocephalus soudanensis*) [87]; *mt-seqs* for five South American electric fishes (*Eigenmannia virescens*, *Apteronotus albifrons*, *Electrophorus electricus*, *Gymnorhamphichthys hypostomus*, and *Gymnotus carapo*) [85], [125]; and a selection of 43 *mt-seqs* for other teleost species. The *mt-seq* of *Amia calva* (Amiiformes) was used to root the tree. Table S1 provides additional information on the specimens included in our study, along with accession numbers for *mt-seq* data archived in the DDBJ, EMBL, and GenBank databases.

Lavoué et al. [119] give detailed descriptions of the standard laboratory protocols for the long and short PCR reactions that were used to obtain complete *mt-seqs* from 95% ethanol-preserved fin and muscle tissue. Complete lists of primer sequences and cycling conditions for these PCR reactions are available upon request to SL.

### Sequence Quality and Alignment

Individual sequences were checked for quality and concatenated to assemble a consensus sequence for each mitogenome using the Sequencher software package v.4.8 (Gene Codes). Gene content and order of the newly determined *mt-seqs* are typical of those found in most other teleosts. Consensus sequences were then exported for analyses using other software.

Across the 70 species considered herein, sequences at each protein-coding gene were aligned manually with respect to the translated amino acid sequence. We excluded the heterogeneous base composition ND6 gene, short ambiguous stretches of alignment at the 5′ and 3′ ends of some protein-coding genes, and all stop codons from subsequent phylogenetic analyses. The 12 S and 16 S rRNA sequences, as well as the concatenated 22 tRNA genes, were aligned with the software Proalign v.0.5 [129] using default parameter settings. Regions with posterior probabilities ≤50% were excluded from the subsequent analyses. The aligned and conditioned data matrix included 14,447 nucleotide positions in total.

### Partitioned Maximum Likelihood Phylogenetic Inference

We inferred phylogenetic trees by means of partitioned maximum likelihood (ML) using the software RAxML [130] with its graphical interface, raxmlGUI 0.9Beta3 [131]. Three different *mt-seq* data subsets, each prepared from the full data matrix, were employed for this purpose. The first data subset (“12RT,” 10,816 positions) included concatenated nucleotide sequences from 22 transfer RNA genes (1,599 positions) and the two ribosomal RNA genes (1,955 positions) plus the first two codon positions of 12 protein-coding genes (7,262 positions); third codon positions were excluded. The second data subset (“123ryRT,” 14,447 positions) included the same set of characters as the first data subset plus only transversions (not the highly homoplasious transitions) at the third codon positions of protein-coding genes (3,631 positions); limiting the analysis to transversions was implemented by replacing third position thymines (T) and cytosines (C) with “Y,” and replacing third position guanines (G) and adenines (A) with “R.” The third data subset (“123RT,” 14,447 positions) included all positions and types of substitution. We set up three partitions (data subset “12RT”: first and second codon positions of protein-coding genes, and all positions of non-protein-coding genes [tRNAs+rRNAs]) or four partitions (data subsets “123ryRT” and “123RT”: same three partitions as for data subset “12RT” plus the third codon positions of protein-coding genes).

According to analyses done using MEGA 5.05 [132], the GTR + Γ + I model–the general time reversible model with discrete gamma distributed rate heterogeneity with allowance for a proportion of invariant sites [133]–was found to be the “best” model of sequence evolution for each partition of our dataset. However, following the cautionary remarks of Stamakis in the RAxML user’s manual [130], as well as those of Yang [134], we did not allow for invariant sites in our model. Instead, we simplified it to the GTR + Γ model as recommended by Stamakis. We performed ML heuristic phylogenetic searches under this model, with data partitioning as described above. One hundred searches were made for each of the three analyses, and the best ML tree was found in each case by comparing final likelihoods among all 100 inferred trees. To evaluate the robustness of the internal branches of each of the best ML trees, 500 bootstrap replicates were calculated for each data subset under the GTR + Γ model. We then visually assessed congruence of the tree topologies resulting from the analyses we performed using the three different data subsets.

### Bayesian Phylogenetic Inference and Divergence Time Estimation

We simultaneously inferred phylogenetic trees and divergence times with 95% credibility intervals. We did so using a partitioned Bayesian method that incorporated a relaxed molecular clock, as implemented by the software BEAST v.1.6.1 [135] and its suite, including BEAUTi v.1.6.1, LogCombiner v.1.6.1, and TreeAnnotator v.1.6.1. BEAUTi was first used to build the XML input files.

Mitochondrial substitution saturation can severely mislead divergence time estimation when not corrected (e.g. [136]–[138]). However, the negative effects of saturation are significantly reduced by removing the highly saturated third positions and using an appropriate system to partition coding and non-coding sequences and first and second codon positions [136], [137]. We therefore restricted our divergence time analysis to the data subset “12RT.” This data subset excludes third codon positions and partitions the remaining two positions separately from a third partition erected for non-protein-coding genes. The GTR + Γ model of sequence evolution was chosen for each of the three data partitions, and parameters were unlinked between partitions. In BEAUTi, we defined 17 taxon subsets for which we constrained their respective ages (based on fossil records) to follow either a lognormal distribution or a uniform distribution, as explained below.

For each reconstruction, two independent runs of 1×10^8^ generations each were performed using BEAST. Each run was initiated from a user-starting chronogram tree that we previously built with BEAST and the data subset “12RT” using a simple HKY model of sequence evolution, a single partition, a strict molecular clock, and a single prior age constraint for the root of the tree set at 284 Mya [95]. In each run, trees and divergence time estimates were sampled once every 5,000 generations, and each run’s parameters were checked for convergence with the software Tracer v.1.5 [135]. We also graphically determined appropriate burn-in periods for each run (at least 10% of total run length). After discarding the burn-in, the remaining tree samples from the two runs were pooled into a combined file using Logcombiner. Finally, we used TreeAnnotator to calculate the maximum clade credibility tree, along with posterior probability support and mean divergence times and their 95% credibility intervals.

### Fossil Selection and Calibration Strategy

Estimates of divergence time based on molecular evidence and a relaxed molecular clock are strongly dependent on how a phylogenetic tree is calibrated by selected fossils. We used two different strategies to calibrate our chronogram. Each method was based on different assumptions regarding the adequacy of the fossil record for estimating maximum ages of ancient teleost groups. Applying both of these methods in a single study allowed us to evaluate how absolute estimates for the ages of the Gymnotiformes and the Mormyroidea vary depending on calibration constraint methodology.

The first strategy we applied (reconstruction #1), which yielded more conservative (i.e., younger) age estimates, assumed that while the fossil records of some groups of Teleostei are informative only to provide minimum age limits for some nodes on the tree, the fossil records of other groups are sufficiently rich to also be informative about maximum ages [139]. Under this strategy, prior age distributions of fossil-calibrated nodes are mixed: either they are assumed to follow a uniform distribution, in which the minimum age limit is equal to the minimum age of the stratum from which the fossil was excavated and the maximum age limit is equal to the minimum age of the root of our tree, or a lognormal distribution, with a minimum age equal to the minimum age of the stratum and a maximum age equal to the maximum age of the stratum.

The second calibration strategy (reconstruction #2) assumes that the overall fossil record of the Teleostei is so incomplete that it provides only minimum ages for the origins of selected groups [86], [96]. In this case, prior age distributions of selected nodes follow a uniform distribution in which the minimum age limit is equal to the minimum age of the stratum from which the fossil was excavated, and the maximum age limit is equal to the minimum age of the root of our tree.

For both reconstructions, we selected 17 fossils in total that we further divided in two sets under reconstruction #1. The first set is composed of seven key fossils deemed to provide the best information about both minimum and maximum age constraints. The second set is composed of ten additional fossils that are less informative and only provide minimum age constraints.

The first six fossils of the first set belong to basal teleost groups having rich and coherent fossil records, while the seventh fossil represents a non-teleost group. Specifically, the seven fossils composing the first set are the following: (1) †*Baugeichthys caeruleus* (Albuliformes) provided a calibration for the time of the most recent common ancestor (tMRCA) for the clade (Anguilliformes, Albuliformes); (2) †*Anaethalion* spp. (Elopiformes) provided a calibration point for the crown-group Elopomorpha; (3) †*Atolvorator longipectoralis* (Aulopiformes) provided a calibration point for the crown-group Euteleostei; (4) †*Laeliichthys australis* (related to *Arapaima gigas* and *Heterotis niloticus*) constrained the age of the crown-group Osteoglossidae; (5) †*Yanbiania wangqingica* (Hiodontiformes) provided a calibration for the crown-group Osteoglossomorpha; (6) †*Tischlingerichthys viohli* (a stem-group ostariophysan) constrained the age of the crown-group Otocephala; and (7) †*Brachydegma caelatum* (Amiiformes) constrained the age of the stem-group Teleostei.

The remaining ten fossils, which compose the second set, are the following: (8) †*Santanichthys diasii* provided a minimum age for the most recent common ancestor (MRCA) of the clade (Siluriformes, Characiformes), i.e. excluding the Gymnotiformes; (9) †*Rubiesichthys gregalis* provided a minimum age for the MRCA of the crown group Ostariophysi; (10) †*Chanos leopoldi* provided a minimum age for the MRCA of the clade (*Chanos*, (*Phractolaemus*, *Grasseichthys*); (11) the oldest bagrids (†*Eomacrones wilsoni*, †*Nigerium gadense*, †*Nigerium wurnoënse*) and the oldest ictalurid (†*Astephus* sp.) provided a minimum age for the MRCA of the clade (*Pseudobagrus*, *Ictalurus*); (12) †*Estesesox foxi* from the Campanian provided a minimum age for the MRCA of the clade (*Esox*, *Salmo*); (13) †*Hoplopteryx* and †*Trachichthyoides* from the Cenomanian provided a minimum age for the MRCA of the clade (*Gadus*, *Paralichthys*); (14) a fossil †*Scleropages* sp. provided a minimum age for the MRCA of the clade (*Scleropages*, *Osteoglossum*); (15) †*Paradercetis kipalaensis* provided a minimum age for the MRCA of the clade (*Arapaima*, *Heterotis*); (16) †*Elopoides* provided a minimum age for the MRCA of the clade (*Elops*, *Megalops*); finally (17) †*Carpathichthys polonicus* provided a minimum age for the clade (*Alepocephalus*, *Platytroctes*).

Details on the origins of these fossil specimens, justifications for their use in calibrating minimum (and sometimes maximum) ages for nodes on the tree, relevant references, and the resulting minimum and maximum age calibrations themselves are provided in Text S1. In making these fossil-based calibrations, we followed ages of the geological stages determined by the international stratigraphy chart of the International Commission on Stratigraphy of 2009 (available online at http://www.stratigraphy.org/).

## Supporting Information

Figure S1 Best maximum likelihood tree of the Teleostei from analysis of the *mt-seq* data subset “123ryRT,” using the software RAxML. Branch lengths are proportional to the number of substitutions per nucleotide position (scale bar  = 0.04 substitutions). Numbers at nodes give node support in terms of bootstrap proportions. The tree is rooted with *Amia calva*. Light grey gradient boxes highlight the Mormyroidea (African weakly electric fishes) and the Gymnotiformes (South American weakly electric fishes). Arrowheads indicate nodes for which topological differences were found compared to trees reconstructed using the two other data subsets (“12RT” and “123RT,” shown in Fig. 3 and S2, respectively).
(EPS)

Figure S2 Best maximum likelihood tree of the Teleostei from analysis of the *mt-seq* data subset “123RT,” using the software RAxML. Branch lengths are proportional to the number of substitutions per nucleotide position (scale bar  = 0.2 substitutions). Numbers at nodes give node support in terms of bootstrap proportions. The tree is rooted with *Amia calva*. Light grey gradient boxes highlight the Mormyroidea (African weakly electric fishes) and the Gymnotiformes (South American weakly electric fishes). Arrowheads indicate nodes for which topological differences were found compared to trees reconstructed using the two other data subsets (“12RT” and “123ryRT,” shown in Fig. 3 and S1, respectively).
(EPS)

Table S1 List of the 70 species included in this study.
(DOCX)

Text S1 Fossil selection for chronogram node calibration.
(DOCX)
