# Supplementary material for: Comparable Ages for the Independent Origins of Electrogenesis in African and South American Weakly Electric Fishes
Source: PLoS One. 2012 May 14;7(5):e36287. doi: 10.1371/journal.pone.0036287 (PMC3351409; doi:10.1371/journal.pone.0036287)
Supplement: Table S1 — List of the 70 species included in this study. (DOCX) [file pone.0036287.s003.docx]

**Table S1.** **List of the 70 species included in this study**. Classification follows Wiley and Johnson [[1](#_ENREF_1)] with the exception of the Cohort Otocephala, which groups Clupeiformes, Gonorynchiformes, Otophysi (*i.e.* Cypriniformes, Characiformes, Siluriformes and Gymnotiformes) and Alepocephaliformes [[2](#_ENREF_2),[3](#_ENREF_3)]. Green boxes indicate electroreceptive and electrogenic fishes, and blue boxes indicate electroreceptive fishes which are not electrogenic.

| **Classification** | Species | Origin | Accession Nos. | Reference |
| --- | --- | --- | --- | --- |
| Order Amiiformes | *Amia calva* Linnaeus 1766 | North America | AB042952 | Inoue et al. [[4](#_ENREF_4)] |
| **Cohort Osteoglossomorpha** |  |  |  |  |
| Family Hiodontidae | *Hiodon alosoides* (Rafinesque 1819) | North America | AP004356 | Inoue et al. [[4](#_ENREF_4)] |
|  | *Hiodon tergisus* Lesueur 1818 | North America | AP009499 | Lavoué et al. [[5](#_ENREF_5)] |
| Family Osteoglossidae | *Osteoglossum bicirrhosum* (Cuvier 1829) | South America | AB043025 | Inoue et al. [[6](#_ENREF_6)] |
|  | *Scleropages formosus* (Müller & Schlegel 1844) | Southeast Asia | DQ023143 | Yue et al. [[7](#_ENREF_7)] |
|  | *Heterotis niloticus* (Cuvier 1829) | Africa | AP009498 | Lavoué et al. [[5](#_ENREF_5)] |
|  | *Arapaima gigas* (Schinz 1822) | South America | AP009497 | Lavoué et al. [[5](#_ENREF_5)] |
| Family Pantodontidae | *Pantodon buchholzi* Peters 1876 | Niger R., Nigeria | AB043068 | Inoue et al. [[6](#_ENREF_6)] |
|  | *Pantodon buchholzi* Peters 1876 | Odzala, Congo basin | AP011564 | Lavoué et al. [[5](#_ENREF_5)] |
| Family Notopteridae | *Xenomystus nigri* (Günther 1868) | Ouémé R., Bénin | AP009503 | Lavoué et al. [[5](#_ENREF_5)] |
| Family Gymnarchidae | *Gymnarchus niloticus* Cuvier 1829 | Ouémé R., Bénin | AP009610 | Lavoué et al. [[5](#_ENREF_5)] |
| Family Mormyridae | *Petrocephalus soudanensis* Bigorne & Paugy 1990 | Ouémé R., Bénin | AP009502 | Lavoué et al. [[5](#_ENREF_5)] |
|  | *Petrocephalus microphthalmus* Pellegrin 1908 | Ivindo R., Gabon | AP009609 | Lavoué et al. [[5](#_ENREF_5)] |
|  | *Myomyrus macrops* Boulenger 1914 | Odzala, Congo basin | AP009501 | Lavoué et al. [[5](#_ENREF_5)] |
|  | *Mormyrops anguilloides* (Linnaeus 1758) | Ouémé R., Bénin | AP011576 | **This study** |
|  | *Isichthys henryi* Gill 1863 | Ivindo R., Gabon | AP011573 | **This study** |
|  | *Brienomyrus brachystius* (Gill 1862) | Ouémé R., Bénin | AP011569 | **This study** |
|  | *Mormyrus rume* Valenciennes 1847 | Ouémé R., Bénin | AP011577 | **This study** |
|  | *Stomatorhinus cf. ater* Pellegrin 1924 | Odzala, Congo basin | AP011583 | **This study** |
|  | *Hyperopisus bebe* (Lacepède 1803) | Ouémé R., Bénin | AP011572 | **This study** |
|  | *Pollimyrus adspersus* (Günther 1866) | Ouémé R., Bénin | AP011582 | **This study** |
|  | *Ivindomyrus marchei* (Sauvage 1879) | Ivindo R., Gabon | AP011574 | **This study** |
|  | *Boulengeromyrus knoepffleri* Taverne & Géry 1968 | Ivindo R., Gabon | AP011568 | **This study** |
|  | *Brevimyrus niger* (Günther 1866) | Ouémé R., Bénin | AP009612 | **This study** |
|  | *Paramormyrops* sp. 'type 2' (magnostipes complex) | Ivindo R., Gabon | AP011578 | **This study** |
|  | *Paramormyrops gabonensis* Taverne, Thys van den Audenaerde & Heymer 1977 | Ivindo R., Gabon | AP009614 | **This study** |
|  | *Marcusenius senegalensis* (Steindachner 1870) | Ouémé R., Bénin | AP011575 | **This study** |
|  | *Gnathonemus petersii* (Günther 1862) | Odzala, Congo basin | AP009611 | Lavoué et al. [[5](#_ENREF_5)] |
|  | *Campylomormyrus numenius* (Boulenger 1898) | Aquarium import | AP011571 | **This study** |
|  | *Cyphomyrus discorhynchus* (Peters 1852) | Odzala, Congo basin | AP009613 | **This study** |
|  | *Genyomyrus donnyi* Boulenger 1898 | Odzala, Congo basin | AP009500 | **This study** |
| **Cohort Elopomorpha** |  |  |  |  |
| Family Elopidae | Elops hawaiensis **Regan 1909** | West central Pacific | AB051070 | Inoue et al. [[8](#_ENREF_8)] |
| Family Megalopidae | Megalops atlanticus **Valenciennes 1847** | Atlantic | AP004808 | Inoue et al. [[8](#_ENREF_8)] |
| Family Notacanthidae | Notacanthus chemnitzii **Bloch 1788** | Marine worldwide | AP002975 | Inoue et al. [[4](#_ENREF_4)] |
| Family Anguillidae | *Anguilla japonica* **Temminck & Schlegel 1846** | Northwest Pacific | AB038556 | Inoue et al. [[9](#_ENREF_9)] |
| Family Muraenidae | Gymnothorax kidako **(Temminck & Schlegel 1846)** | Indo-Pacific | AP002976 | Inoue et al. [[4](#_ENREF_4)] |
| **Cohort Otocephala** |  |  |  |  |
| Order Clupeiformes |  |  |  |  |
| Family Denticipitidae | Denticeps clupeoides **Clausen 1959** | West Africa | AF007276 | Lavoué et al. [[10](#_ENREF_10)] |
| Family Clupeidae | Sardinops melanostictus **(Temminck & Schlegel 1846)** | Northwest Pacific | AB032554 | Inoue et al. [[11](#_ENREF_11)] |
| Family Engraulidae | Engraulis Japonicus **Temminck & Schlegel 1846** | Northwest Pacific | AB040676 | Inoue et al. [[12](#_ENREF_12)] |
|  | *Coilia nasus* **Temminck & Schlegel 1846** | Northwest Pacific | AP009135 | Lavoué et al. [[13](#_ENREF_13)] |
| Family Pristigasteridae | *Ilisha elongata* **(Anonymous [Bennett] 1830)** | Indo-Pacific | AP009141 | Lavoué et al. [[13](#_ENREF_13)] |
|  | *Ilisha africana* **(Bloch 1795)** | East Atlantic | AP009140 | Lavoué et al. [[13](#_ENREF_13)] |
| Family Sundasalangidae | *Sundasalanx mekongensis* **Britz & Kottelat 1999** | Mekong R, Cambodia | AP006232 | Ishiguro et al. [[14](#_ENREF_14)] |
| Order Alepocephaliformes |  |  |  |  |
| Family Alepocephalidae | *Alepocephalus tenebrosus* **Gilbert 1892** | Indo-Pacific | AP004100 | Ishiguro et al. [[2](#_ENREF_2)] |
| Family Platytroctidae | *Platytroctes apus* **Günther 1878** | Indo-Pacific | AP004107 | Ishiguro et al. [[2](#_ENREF_2)] |
| Order Gonorynchiformes |  |  |  |  |
| Family Chanidae | Chanos chanos **(Forsskål 1775)** | Indo-Pacific | AB054133 | Saitoh et al. [[15](#_ENREF_15)] |
| Family Gonorynchidae | *Gonorynchus greyi* **(Richardson 1845)** | marine Australia | AB054134 | Ishiguro et al. [[2](#_ENREF_2)] |
| Family Phractolaemidae | *Phractolaemus ansorgii* **Boulenger 1901** | West Africa | AF007280 | Lavoué et al. [[10](#_ENREF_10)] |
| Family Kneriidae | Grasseichthys gabonensis **Géry 1964** | Ivindo R., Gabon | AF007277 | Lavoué et al. [[10](#_ENREF_10)] |
| Order Cypriniformes |  |  |  |  |
| Family Cyprinidae | Carassius auratus **(Linnaeus 1758)** | Eurasia | AB006953 | Murakami et al. [[16](#_ENREF_16)] |
| Family Cobitidae | Cobitis striata **Ikeda 1936** | Japan | AB054125 | Saitoh et al. [[15](#_ENREF_15)] |
|  | Lefua echigonia **Jordan & Richardson 1907** | Japan | AB054126 | Saitoh et al. [[15](#_ENREF_15)] |
| Family Balitoridae | Crossostoma lacustre **Steindachner 1908** [=*Formosania lacustris*] | Taiwan | M91245 | Tzeng et al. [[17](#_ENREF_17)] |
| Order Characiformes |  |  |  |  |
| Family Characidae | Phenacogrammus interruptus **(Boulenger 1899)** | West Africa | AB054129 | Saitoh et al. [[15](#_ENREF_15)] |
|  | *Chalceus macrolepidotus* **Cuvier 1818** | South America | AB054130 | Saitoh et al. [[15](#_ENREF_15)] |
| Order Gymnotiformes |  |  |  |  |
| Family Sternopygidae | Eigenmania virescens **(Valenciennes 1836)** | South America | AB054131 | Saitoh et al. [[15](#_ENREF_15)] |
| Family Apteronotidae | Apteronotus albifrons **(Linnaeus 1766)** | South America | AB054132 | Saitoh et al. [[15](#_ENREF_15)] |
| Family Gymnotidae | *Electrophorus* *electricus* **(Linnaeus 1766)** | South America | AP011978 | Nakatani et al. [[18](#_ENREF_18)] |
|  | *Gymnotus* *carapo* **Linnaeus 1758** | South America | AP011979 | Nakatani et al. [[18](#_ENREF_18)] |
| Family Hypopomidae | *Brachyhypopomus* *occidentalis* **(Regan 1914)** | South America | AP0011570 | **This study** |
| Family Rhamphichthyidae | *Gymnorhamphichthys* *hypostomus* **Ellis 1912** | South America | AP011980 | Nakatani et al. [[18](#_ENREF_18)] |
| Order Siluriformes |  |  |  |  |
| Family Bagridae | Pseudobagrus tokiensis **Döderlein 1887** | Southeast Asia | AB054127 | Saitoh et al. [[15](#_ENREF_15)] |
| Family Callicthyidae | Corydoras rabauti **La Monte 1941** | South America | AB054128 | Saitoh et al. [[15](#_ENREF_15)] |
| Family Ictaluridae | *Ictalurus punctatus* (Rafinesque 1818) | North America | AF482987 | Waldbieser et al. [[19](#_ENREF_19)] |
| **Cohort Euteleostei** **(=Euteleosteomorpha)** |  |  |  |  |
| Subcohort Protacanthopterygii |  |  |  |  |
| Family Salmonidae | Coregonus lavaretus **(Linnaeus 1758)** | Europe | AB034824 | Miya & Nishida [[20](#_ENREF_20)] |
| Family Esocidae | Esox lucius **Linnaeus 1758** | Eurasia, North America | AP004103 | Ishiguro et al. [[2](#_ENREF_2)] |
| Subcohort Neoteleostei |  |  |  |  |
| Family Gadidae | Gadus morhua **Linnaeus 1758** | North Atlantic | X99772 | Johansen & Bakke [[21](#_ENREF_21)] |
| Family Paralichthyidae | Paralichthys olivaceus **(Temminck & Schlegel 1846)** | Northwest Pacific | AB028664 | Saitoh et al. [[22](#_ENREF_22)] |

**References**

1. Wiley EO, Johnson GD (2010) A teleost classification based on monophyletic groups. In: Nelson JS, Schultze HP, Wilson MVH, editors. Origin and phylogenetic interrelationships of teleosts. München: Verlag Dr. Friedrich Pfeil. pp 123-182.

2. Ishiguro NB, Miya M, Nishida M (2003) Basal euteleostean relationships: a mitogenomic perspective on the phylogenetic reality of the "Protacanthopterygii". Mol Phylogenet Evol 27: 476-488.

3. Lavoué S, Miya M, Poulsen JY, Moller PR, Nishida M (2008) Monophyly, phylogenetic position and inter-familial relationships of the Alepocephaliformes (Teleostei) based on whole mitogenome sequences. Mol Phylogenet Evol 47: 1111-1121.

4. Inoue JG, Miya M, Tsukamoto K, Nishida M (2003) Basal actinopterygian relationships: a mitogenomic perspective on the phylogeny of the "ancient fish". Mol Phylogenet Evol 26: 110-120.

5. Lavoué S, Miya M, Arnegard ME, McIntyre PB, Mamonekene V, et al. (2011) Remarkable morphological stasis in an extant vertebrate despite tens of millions of years of divergence. Proc R Soc London B 278: 1003-1008.

6. Inoue JG, Miya M, Tsukamoto K, Nishida M (2001) A mitogenomic perspective on the basal teleostean phylogeny: resolving higher-level relationships with longer DNA sequences. Mol Phylogenet Evol 20: 275-285.

7. Yue GH, Liew WC, Orban L (2006) The complete mitochondrial genome of a basal teleost, the Asian arowana (*Scleropages formosus*, Osteoglossidae). BMC Genomics 7: 242.

8. Inoue JG, Miya M, Tsukamoto K, Nishida M (2004) Mitogenomic evidence for the monophyly of elopomorph fishes (Teleostei) and the evolutionary origin of the leptocephalus larva. Mol Phylogenet Evol 32: 274-286.

9. Inoue JG, Miya M, Aoyama J, Ishikawa S, Tsukamoto K, et al. (2001) Complete mitochondrial DNA sequence of the Japanese eel *Anguilla japonica*. Fish Sci 67: 118-125.

10. Lavoué S, Miya M, Inoue JG, Saitoh K, Ishiguro N, et al. (2005) Molecular systematics of the gonorynchiform fishes (Teleostei) based on whole mitogenome sequences: Implications for higher-level relationships within the Otocephala. Mol Phylogenet Evol 37: 165-177.

11. Inoue JG, Miya M, Tsukamoto K, Nishida M (2000) Complete mitochondrial DNA sequence of the Japanese sardine *Sardinops melanostictus*. Fish Sci 66: 924-932.

12. Inoue JG, Miya M, Tsukamoto K, Nishida M (2001) Complete mitochondrial DNA sequence of the Japanese anchovy *Engraulis japonicus*. Fish Sci 67: 828-835.

13. Lavoué S, Miya M, Saitoh K, Ishiguro NB, Nishida M (2007) Phylogenetic relationships among anchovies, sardines, herrings and their relatives (Clupeiformes), inferred from whole mitogenome sequences. Mol Phylogenet Evol 43: 1096-1105.

14. Ishiguro NB, Miya M, Inoue JG, Nishida M (2005) *Sundasalanx* (Sundasalangidae) is a progenetic clupeiform, not a closely-related group of salangids (Osmeriformes): mitogenomic evidence. J Fish Biol 67: 561-569.

15. Saitoh K, Miya M, Inoue JG, Ishiguro NB, Nishida M (2003) Mitochondrial genomics of ostariophysan fishes: perspectives on phylogeny and biogeography. J Mol Evol 56: 464-472.

16. Murakami M, Yamashita Y, Fujitani H (1998) The complete sequence of mitochondrial genome from a gynogenetic triploid "ginbuna" (*Carassius auratus langsdorfi*). Zool Sci 15: 335-337.

17. Tzeng C-S, Hui C-F, Shen S-C, Huang PC (1992) The complete nucleotide sequence of the *Crossostoma* *lacustre* mitochondrial genome: conservation and variations among vertebrates. Nuc Acids Res 20: 4853-4858.

18. Nakatani M, Miya M, Mabuchi K, Saitoh K, Nishida M (2011) Evolutionary history of Otophysi (Teleostei), a major clade of the modern freshwater fishes: Pangaean origin and Mesozoic radiation. BMC Evol Biol 11: e177.

19. Waldbieser GC, Bilodeau AL, Nonneman DJ (2003) Complete sequence and characterization of the channel catfish mitochondrial genome. DNA Sequence 14: 265-277.

20. Miya M, Nishida M (2000) Use of mitogenomic information in teleostean molecular phylogenetics: A tree-based exploration under the maximum-parsimony optimality criterion. Mol Phylogenet Evol 17: 437-455.

21. Johansen S, Bakke I (1996) The complete mitochondrial DNA sequence of Atlantic cod (*Gadus morhua*): Relevance to taxonomic studies among codfishes. Molecular Mar Biol Biotechnol 5: 203-214.

22. Saitoh K, Hayashizaki K, Yokoyama Y, Asahida T, Toyohara H, et al. (2000) Complete nucleotide sequence of Japanese flounder (*Paralichthys olivaceus*) mitochondrial genome: Structural properties and cue for resolving teleostean relationships. J Hered 91: 271-278.
