# Supplementary material for: Comparable Ages for the Independent Origins of Electrogenesis in African and South American Weakly Electric Fishes
Source: PLoS One. 2012 May 14;7(5):e36287. doi: 10.1371/journal.pone.0036287 (PMC3351409; doi:10.1371/journal.pone.0036287)
Supplement: Text S1 — Fossil selection for chronogram node calibration. (DOCX) [file pone.0036287.s004.docx]

**Text S1. Fossil selection for chronogram node calibration**

**First set of fossils providing minimum ages under both calibration strategies (and maximum ages under the first calibration strategy; i.e. reconstruction #1):**

1- The Albuliformes, including the Notacanthiformes, have a relatively rich fossil record [1]. In our study, we constrained the age of the most recent common ancestor (MRCA) of the clade (Anguilliformes, Albuliformes) to a minimum of 130 million years ago (Mya) and a maximum of 134 Mya [lognormal distribution mean = 1; standard deviation (stdev) = 0.5]. This age constraint corresponds to the age of the oldest albuliform fossil known, †*Baugeichthys caeruleus* [2], from the Hauterivian stage (see also Fig. 1 in Mayrinck et al. [1]). The fossil record of the order Anguilliformes, the sister group of the Albuliformes, is also rather informative. Although the first anguilliform fossils are only of Cenomanian age (99.6-93.6 Mya), they demonstrate that the Anguilliformes had already undergone significant radiation in form by this time [3,4], suggesting an origin earlier than the Cenomanian.

2- †*Anaethalion* spp. from the Kimmeridgian age of the Late Jurassic epoch (155.6-150.8 Mya) are the earliest elopomorph fossils in a long series of fossils that gives good information on the origins of the Elopiformes and the Elopomorpha in the Mesozoic era, as well as their subsequent diversification up to the present [5]. Members of this extinct genus are considered to be the oldest crown-group teleost fossils [5]. Thus, we used †*Anaethalion* spp. to calibrate the age of the crown-group Elopomorpha to a minimum age of 150.8 Mya and a maximum age of 155.6 Mya [lognormal distribution mean = 1.4; stdev = 0.6].

3- Davis and Fielitz [6] gave an updated account of the fossil record of the Aulopiformes, in which they listed no less than 31 extinct taxa; the oldest known, †*Atolvorator longipectoralis* [7], dates back to the Barremian age (130-125 Mya). We constrained the age of MRCA of the crown-group Euteleostei using this most ancient aulopiform fossil. The phylogenetic position of this fossil is still unresolved within the Aulopiformes, and it could potentially represent a somewhat derived form. Nevertheless, because older material has not been discovered in the Aulopiformes and crown-group Euteleostei, we used †*A. longipectoralis* to constrain the minimum age of the crown-group Euteleostei to 125 Mya and its maximum age to 130 Mya [lognormal distribution mean = 1; stdev = 0.5].

4- Forey and Hilton [8] provided a critical review of the rather informative fossil record of the family Osteoglossidae (including at least 29 named fossil taxa and 11 unnamed fossil specimens) in strata deposited from the Early Cretaceous period to the Neogene period. This collection of fossils is quite significant for understanding the history of the Osteoglossidae [8], although uncertainty about the phylogenetic position of several component fossils limits their suitability for divergence time calibration. Whereas all extant osteoglossid species are strictly freshwater fishes, several fossils come from marine deposits. Based on these data, Bonde [9] and Forey and Hilton [8] concluded that the seemingly-simple biogeography of the Osteoglossidae based on extant taxa (e.g., with vicariant origins via the breakup of Pangea) may have been much less simple than this, in fact. Despite these complexities, there is broad agreement about the placement of extant osteoglossids in the only two constituent subfamilies, Osteoglossinae (*Scleropages*, *Osteoglossum*) and Heterotidinae (*Arapaima*, *Heterotis*) [10,11]. Given this context, we constrained the MRCA of the crown-group Osteoglossidae to a minimum age of 112 Mya and a lower 95% confidence interval (i.e., maximum age) of 125 Mya [lognormal distribution mean = 2.3; stdev = 0.5] on the basis of †*Laeliichthys australis* [10,11,12]. The poorly preserved †*Chandlerichthys* sp. may provide additional supporting evidence for setting our constraint this way ([10,11,12]; but see discussion in Forey and Hilton [8]). Both †*L. australis* and †*Chandlerichthys* sp. are described from South America and are dated to the Aptian age (125-112 Mya); they represent the oldest fossils assigned to the subfamily Heterotidinae (= Arapaiminae).

5- We used †*Yanbiania wangqingica* to calibrate the age of the node of the crown-group Osteoglossomorpha to a minimum of 125 Mya and a lower 95% confidence interval (i.e., maximum age) of 130 Mya [lognormal distribution mean = 0.6; stdev = 0.5]. The first undisputed osteoglossomorph fossils are †*Lycoptera* spp. and †*Yanbiania wangqingica* [13,14]. The genus †*Lycoptera* is a stem-group osteoglossomorph. In contrast, †*Yanbiania* *wangqingica* is considered to be the oldest fossil unambiguously assigned to the crown-group Osteoglossomorpha; it has been placed within the order Hiodontiformes [13]. †*Yanbiania* *wangqingica* is dated to the Barremian age (130-125 Mya) [10,15].

6- The age of the MRCA of the Otocephala was set to 155.6-150.8 Mya [lognormal distribution mean = 1.4; stdev = 0.6] on the basis of the oldest representative fossil known, †*Tischlingerichthys viohli*, which is a stem-group ostariophysan from the Tithonian stage (uppermost stage of the Upper Jurassic series) [5,16,17]. As Benton [17] stated, the fossil record of the sister group of the Otocephala, the Euteleostei, is in agreement with this result, with two stem-group euteleosts collected from the same stratum (†*Leptolepides*, †*Orthogonikleithrus*).

7- Finally, the age of the MRCA of the clade composed of *Amia calva* and the Teleostei (i.e. the root of our tree) was set to a minimum of 284.4 Mya and a maximum of 311.7 Mya [lognormal distribution mean = 2 and stdev = 0.5]. The first date corresponds to the upper boundary of the Sakmarian stage in the Permian system, from which the oldest known amiiform fossil, †*Brachydegma*, was excavated [18]. Hurley et al. [18] suggested that †*Brachydegma* provides a solid basis for setting the minimum age of the stem-group Teleostei, even if the most ancient stem-group teleost fossil is only 225 My old. The second date (i.e., maximum age) corresponds to the oldest stem group Neopterygii, †*Discoserra* [18].

**Second set of fossils providing only minimum ages under both calibration strategies:**

8- We used †*Santanichthys diasii* [19] to calibrate the minimum age of the MRCA of the clade (Siluriformes, Characiformes)—i.e., excluding the Gymnotiformes—to 125 Mya. Filleul and Maisey [19] redescribed †*S. diasii* from the Barremian stage, identifying this fossil as a stem characiform species. As such, it represents the oldest known characiform fossil.

9- The oldest fossil placed in the order Gonorynchiformes is †*Rubiesichthys gregalis*, a stem gonorynchiform [20] collected from the Berriasian stage of the Lower Cretaceous series (145.5-140.2 Mya). This fossil was used to provide the minimum age of the Gonorynchiformes and the Otophysi, i.e., the minimum age of the crown group Ostariophysi (=140.2 Mya).

10- The earliest fossil unambiguously assigned to the crown group Gonorynchiformes is a species of *Chanos* (family Chanidae), †*Chanos* *leopoldi*, collected from the Aptian stage of the Lower Cretaceous series (corresponding to the time period from 125-112 Mya) [20]. We used this fossil to constrain the minimum age of the MRCA of the clade (*Chanos*, (*Phractolaemus*, *Grasseichthys*)) to 112 Mya.

11- The first siluriform fossil may be an undescribed species collected from Coniacian-Santonian strata in Africa [21,22]. In slightly higher strata, several siluriform fossils begin to appear in Campanian and Maastrichtian stages in South America [22]. The oldest bagrids (†*Eomacrones wilsoni*, †*Nigerium gadense*, and †*Nigerium wurnoënse*) and the oldest ictalurid (†*Astephus* sp.) are found in the Middle Paleocene series, providing a minimum age (58.7 Mya) for the MRCA of the clade (*Pseudobagrus*, *Ictalurus*) [22].

12- The earliest esocoids have been reported from the Early Campanian stage of the Upper Cretaceous series (corresponding to about 83.5 Mya) [23]. Specifically, †*Estesesox foxi* [24] provided us with the minimum age constraint (83.5 Mya) for the MRCA of the clade (*Esox*, *Salmo*).

13- Santini et al. [25] used several beryciform fossils from the Cenomamian age (99.6-93.6 Mya), such as †*Hoplopteryx* sp. and †*Trachichthyoides* sp., to calibrate the age of the MRCA of the Acanthomorpha. In our study, we used these same fossils as grounds for setting the minimum age of the MRCA of the clade (*Gadus*, *Paralichthys*) to 93.6 Mya.

14- In agreement with Taverne et al. [26], Rana [27] described the earliest known fossils that are closely related to the osteoglossomorph genus *Scleropages*; these were collected from the Maastrichthian stage (corresponding to 70.6-65.5 Mya) in the Deccan Traps of India [27]. This fossil evidence provided a minimum age constraint (65.5 Mya) for the clade (*Scleropages*, *Osteoglossum*).

15- Taverne et al. [11] considered †*Paradercetis* *kipalaensis* from the Cenomanian stage in the Democratic Republic of Congo (corresponding to 99.6-93.6 Mya) to be the sister group of *Heterotis niloticus*. They also regarded this extinct taxon to be the most ancient representative of the crown group Arapaiminae [11], which includes two extant genera, *Arapaima* and *Heterotis*. Forey and Hilton [8] did not examine †*P.* *kipalaensis* in their work, though they did discuss previous hypotheses concerning its phylogenetic placement. Given the apparent informativeness of †*P.* *kipalaensis*, we used this fossil to provide a minimum age for the MRAC of the clade (*Arapaima*, *Heterotis*).

16- The earliest stem representatives of the elopiform lineage (with only two living genera, *Elops* and *Megalops*) are as old as the Late Jurassic period, with some representatives being referred to as †*Elops*-like forms [5,28]. This designation refers more to an overall similarity of these extinct †*Elops*-like representatives to the extant genus *Elops*, than to an established close phylogenetic relationship to living *Elops*. Consequently, according to Forey et al. [29], the first crown group elopiform fossil is †*Elopoides*, which is of Barremian age (about 125 Mya). Thus, following Forey et al. [29], we set the minimum age for the MRCA of the clade (*Elops*, *Megalops*) to 125 Mya.

17- The fossil record of the Alepocephaliformes, a deep-sea group of fishes, is sparse. The oldest alepocephaliform fossil is the alepocephalid †*Carpathichthys* *polonicus* of Miocene age [30], which provided us with a minimum age of 23 Mya for the MRCA of the clade (*Alepocephalus*, *Platytroctes*).

**References**

1. Mayrinck D, Brito PM, Otero O (2010) A new albuliform (Teleostei: Elopomorpha) from the Lower Cretaceous Santana Formation, Araripe Basin, northeastern Brazil. Cretaceous Research 31: 227-236.

2. Filleul A (2000) *Baugeichthys caeruleus*, gen. et sp. nov., a new albuliform fish from the Hauterivian of the Massif des Bauges (France). Journal of Vertebrate Paleontology 20: 637-644.

3. Belouze A, Gayet M, Atallah C (2003) The first Anguilliformes: II. Paraphyly of the genus *Urenchelys* WOODWARD, 1900 and phylogenetic relationships. Geobios 36: 351-378.

4. Belouze A, Gayet M, Atallah C (2003) The first Anguilliformes: I. Revision of Cenomanian genera *Anguillavus* HAY, 1903 and *Luenchelys* nov. gen. Geobios 36: 241-273.

5. Arratia G (2000) Remarkable teleostean fishes from the Late Jurassic of southern Germany and their phylogenetic relationships. Fossil Record 3: 137-179.

6. Davis MP, Fielitz C (2010) Estimating divergence times of lizardfishes and their allies (Euteleostei: Aulopiformes) and the timing of deep-sea adaptations. Mol Phylogenet Evol 57: 1194-1208.

7. Gallo V, Coelho PM (2008) First occurrence of an aulopiform fish in the Barremian of the Sergipe-Alagoas Basin, northeastern Brazil. In: Arratia G, Schultze HP, Wilson MVH, editors. Mesozoic Fishes 4-Homology and Phylogeny, Proceedings of the international meeting Miraflores de la Sierra, 2005: Verlag. pp. 351-371.

8. Forey PL, Hilton EJ (2010) Two new Tertiary osteoglossid fishes (Teleostei: Osteoglossomorpha) with notes on the history of the family. In: Elliott DK, Maisey JG, Yu X, Miao D, editors. Morphology, Phylogeny and Paleobiogeography of Fossil Fishes. Munich, Germany: Verlag Dr. Friedrich Pfeil. pp. 215-246.

9. Bonde N (1996) Osteoglossids (Teleostei:Osteoglossomorpha) of the Mesozoic. Comments on their interrelationships. In: Arratia G, Viohl G, editors. Mesozoic Fishes: Systematics and Paleoecology. Munich, Germany: Verlag Dr. Friedrich Pfeil. pp. 273-284.

10. Li G-Q, Wilson MVH (1996) Phylogeny of Osteoglossomorpha. In: Stiassny MLJ, Parenti LR, Johnson GD, editors. Interrelationships of Fishes. New York: Academic press. pp. 163-174.

11. Taverne L (1998) Les ostéoglossomorphes marins de l'Eocène du Monte Bolca (Italie): *Monopteros* Volta, 1796, *Thrissopterus* Heckel, 1856 et *Foreyichthys* Taverne, 1979. Considérations sur la phylogénie des téléostéens ostéoglossomorphes. Studi e Ricerche sui Giacimenti Terziari di Bolca. Verona: Museo Civico di Storia Naturale. pp. 67-158.

12. Li G-Q, Grande L, Wilson MVH (1997) The species of *Phareodus* (Teleostei: Osteoglossidae) from the Eocene of North America and their phylogenetic relationships. J Vert Paleontol 17: 487-505.

13. Wilson MVH, Murray AM (2008) Osteoglossomorpha: phylogeny, biogeography, and fossil record and the significance of key African and Chinese fossil taxa. Geological Society, London, Special Publications 295: 185-219.

14. Zhang JY (2004) New fossil osteoglossomorph from Ningxia, China. J Vert Paleontol 24: 515-524.

15. Murray AM, Wilson MVH (2005) Description of a new Eocene osteoglossid fish and additional information on *Sigida jacksonoides* Greenwood and Patterson, 1967 (Osteoglossomorpha), with an assessmant of their phylogenetic relationships. Zool J Linnean Soc 144: 213-228.

16. Arratia G (1997) Basal teleosts and teleostean phylogeny. Palaeo Ichthyologica 7: 5-168.

17. Benton MJ, Donoghue PCJ, Asher RJ (2009) Calibrating and constraining molecular clocks. In: Hedges SB, Kumar S, editors. The Timetree of Life. New York: Oxford University Press. pp. 35-86.

18. Hurley IA, Mueller RL, Dunn KA, Schmidt EJ, Friedman M, et al. (2007) A new time-scale for ray-finned fish evolution. Proc R Soc London B 274: 489-498.

19. Filleul A, Maisey JG (2004) Redescription of *Santanichthys diasii* (Otophysi, Characiformes) from the Albian of the Santana Formation and comments on its implications for otophysan relationships. Amer Mus Novitates No. 3455: 1-21.

20. Fara E, Gayet M, Taverne L (2010) The fossil record of Gonorynchiformes. In: Grande T, Poyato-Ariza FJ, Diogo R, editors. Gonorynchiformes and Ostariophysan Relationships: A Comprehensive Review. Enfield, New Hampshire, USA: Science Publishers. pp. 173-226.

21. Patterson C (1993) Osteichthyes: Teleostei. In: Benton MJ, editor. The Fossil Record 2. London: Chapman & Hall. pp. 621-656.

22. Gayet M, Otero O (1999) Analysis of the palaeodiversification of the Siluriformes (Osteichthyes, Teleostei, Ostariophysi). Geobios 32: 235-246.

23. Newbrey MG, Wilson MVH, Ashworth AC (2008) Climate change and evolution of growth in Late Cretaceous to Recent North American Esociformes. In: Arratia G, Schultze HP, Wilson MVH, editors. Mesozoic Fishes 4-Homology and Phylogeny. Munich, Germany: Verlag Dr. Friedrich Pfeil. pp. 311-350.

24. Wilson MVH, Brinkman DB, Neuman AG (1992) Cretaceous Esocoidei (Teleostei): early radiation of the pikes in North American fresh waters. J Paleontol 66: 839-846.

25. Santini F, Harmon LJ, Carnevale G, Alfaro ME (2009) Did genome duplication drive the origin of teleosts? A comparative study of diversification in ray-finned fishes. BMC Evol Biol 9: 194.

26. Taverne L, Nolf D, Folie A (2007) On the presence of the osteoglossid fish genus *Scleropages* (Teleostei, Osteoglossiformes) in the continental Paleocene of Hainin (Mons Basin, Belgium). Belg J Zool 137: 89-97.

27. Rana RS (1988) Freshwater fish otoliths from the Deccan Trap associated sedimentary (Cretaceous-Tertiary transition) beds of Rangapur, Hyderabad, District, Andhra Pradesh, India. Geobios 21: 465-493.

28. Arratia G (1987) *Anaethalion* and similar teleosts (Actinopterygii, Pisces) from the Late Jurassic (Tithonian) of Southern Germany and their relationships. Palaeontographica Abteilung A: 1-44.

29. Forey PL, Littlewood DTJ, Ritchie P, Meyer A (1996) Interrelationships of elopomorph fishes. In: Stiassny MLJ, Parenti LR, Johnson GD, editors. Interrelationships of Fishes. New York: Academic press. pp. 175-191.

30. Jerzmanska A (1979) Oligocene alepocephaloid fishes from the Polish Carpathians. Acta Palaeontol Polonica 24: 65-76.
